# Unusual presentation of basilar artery stroke secondary to patent foramen ovale: a case report

**DOI:** 10.1186/1752-1947-2-75

**Published:** 2008-03-07

**Authors:** Abdul Salam, Phil Sanmuganathan, Chris Pycock

**Affiliations:** 1 Specialist Registrar Geriatric/General Medicine, Worcester Royal Hospital, Worcester, WR5 1DD, UK; 2 Consultant Stroke physician, Worcester Royal Hospital, Worcester, WR5 1DD, UK; 3 Consultant Physician, Worcester Royal Hospital, Worcester, WR5 1DD, UK

## Abstract

**Introduction:**

We report a case of a patient presenting with neuropsychiatric manifestations secondary to paradoxical embolism

**Case presentation:**

Unexplained rapid onset of confusion with amnesia and minimal neurological deficits can be a manifestation of various systemic causes of which stroke, either ischemic or hemorrhagic, is one. Thorough and systematic evaluation of these patients can be highly rewarding in terms of optimizing patient outcome. We report the case of a 45-year-old woman whose initial presentation was with acute onset of confusion, memory loss with personality change and headaches. A differential diagnosis of systemic illness and cerebral pathology was entertained. She was empirically treated for neurological infection. Brain imaging revealed bilateral thalamic and cerebellar infarction. Further evaluation with an aim to define the etiology, revealed the diagnosis of Patent Foramen Ovale with paradoxical embolism. The differential diagnosis of unexplained rapid onset of confusion, amnesia with minimal motor neurological deficits and relevant appropriate investigations are discussed in this case report.

**Conclusion:**

This case highlights the importance of recognising the atypical manifestations of posterior fossa stroke. In young patients presenting with non-focal neuropsychiatric manifestations, paradoxical embolism, secondary to patent foramen ovale is a possible cause.

## Introduction

Confusion is a common cause of admission in the older population. Though not as common in younger patients, unexplained rapid onset of confusion with amnesia and minimal neurological deficits can be a manifestation of various systemic causes of which stroke, either ischemic or hemorrhagic, is one. Thorough and systematic evaluation of these patients can be highly rewarding in terms of optimizing patient outcome.

## Case presentation

A 45-year-old woman was admitted with acute onset of short-term memory loss, intermittent headaches, dizzy spells and an obvious change in personality which had progressed over a ten day period. Intermittent vomiting, confusion and unsteadiness were also reported. These symptoms worsened over the three days prior to admission. Her past medical history included an episode of herpes simplex virus infection about one month earlier. A non-smoker, she consumed alcohol in moderation.

On examination, she had disorientation of time and place and her mini mental score was found to be 2/10. Confusion with nominal aphasia was also noted. She was apyrexial. Cardiovascular, respiratory and gastrointestinal system examination was unremarkable. Neurological examination showed normal power and tone in both arms and legs with normal symmetrical deep tendon reflexes. Her gait, coordination, cranial nerve and fundus examination were normal.

Investigations revealed a normal full blood count and unremarkable routine biochemical tests and inflammatory markers. Contrast enhanced CT of the brain showed low attenuation areas adjacent to the anterior ends of both thalami. Appearances were reported as an unusual form of cerebral infarctions and it was suggested that other pathology could not be ruled out.

Examination of CSF revealed isolated lymphocytosis. Glucose and protein were normal. Polymerase Chain Reaction (PCR) tests for herpes simplex virus and varicella zoster were negative.

**Figure 1:**
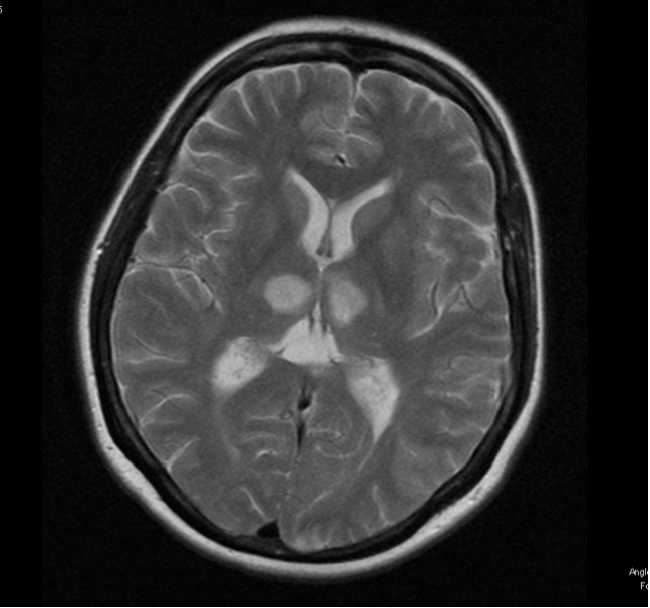
MRI Head showing bilateral symmetrical lesions in both thalami giving high signal changes on T2 weighted images.

Electroencephalogram (EEG) recordings showed intermittent low frequency activity suggesting focal abnormality of cortical function, probably associated with a vascular or structural lesion but no clear evidence of encephalitis or epileptiform activity. On MRI scan (Figure 1) of the head there were bilateral symmetrical lesions in both thalami giving high signal changes on the T2 weighted and proton density images. In addition there were asymmetrical multiple high signal areas on T2 weighted images in both cerebellar hemispheres. CNS lymphoma and demyelination were two of the possible differential diagnoses for the MRI appearances. Subsequently performed vertebral and carotid MR angiograms were reported to be normal and the possibility of basilar artery aneurysm or thrombosis was ruled out.

Over a period of two weeks there was a progressive worsening of confusion, with increasing agitation, memory impairment and personality change. With deterioration in Glasgow Coma Scale (GCS), the possibility of other systemic causes was considered. She was empirically treated for encephalitis and/or viral meningitis and a possibility of vasculitis was considered, for which a trial of methylprednisolone and cyclophosphamide was given without any significant benefit. Further investigations, looking for a possible systemic cause, by an autoantibody screen including anti-neutrophil cytoplasmic antibodies and a vasculitic screen was reported as normal.

A saline contrast transthoracic echo confirmed a diagnosis of Patent Foramen Ovale (PFO) as a significant number of microbubbles appeared in the left atrium within 3 cardiac cycles of their appearance in the right atrium and this was facilitated with valsalva release. Further assessment for atrial septal separation was not done with transesophageal echocardiogram because of the above positive findings from the transthoracic echocardiogram.

A diagnosis of embolic basilar artery ischemic stroke with thalamic and cerebellar infarcts secondary to paradoxical embolism via the PFO was made. She was started on aspirin and clopidogrel and later anticoagulated with warfarin before she underwent percutaneous device closure.

There are three treatment options in patients with a presumed paradoxical embolism and PFO.

1. Antiplatelet therapy with aspirin

2. Oral anticoagulation with warfarin

3. Surgical or percutaneous device closure

Despite the growing evidence for association between PFO and atrial septal aneurysm with paradoxical embolism causing neurological events there is no consensus as how to treat a cryptogenic stroke and none of the above treatments have been evaluated in randomised controlled trials.

There was a gradual improvement over four weeks and subsequently the patient went on to have a complete recovery without any neurological or physical residual effects.

Later she underwent percutaneous endovascular closure of the PFO with a Biostar device. In our patient percutaneous device closure was used because of her young age and absence of deep vein thrombosis or procoagulant state. It was felt inappropriate to leave the patient on life long warfarin with all the risks that anticoagulation might entail. Our patient had further follow up after the device closure of the PFO and had had no further episodes of transient ischaemic attack or any recurrence of stroke.

## Discussion

The incidence of cerebellar infarction in a series of patients with stroke is approximately 1.5% [1]. The thalamus is involved in 25% of all vertebrobasilar strokes, usually in combination with other structures [2]. The commonest cause in patients in younger age groups is arterial occlusion secondary to dissection, then cardio embolic in 27%, either through a patent foramen ovale or due to rheumatic heart disease [3]. Patent foramen ovale is a well-known cause of stroke especially in young patients who have had cryptogenic stroke [4,5]. Patent foramen ovale (PFO), a persistence of an embryonic defect in the interatrial septum, is present in up to 27% of the general population [6]. PFO and atrial septal aneurysms have been identified as potential causative factors of stroke in younger patients when no other etiological cause is found. In our patient we could not find any causative factor including deep vein thrombosis or a cardiac source of embolus, but the causal relation between PFO and stroke was strengthened by the presence of a right to left shunt with a significant sized (5 mm) PFO.

Nater et al reported an increased frequency of posterior circulatory stroke in patients with patent foramen ovale and atrial septal aneurysm [7]. Cardioembolic stroke affecting the posterior circulation is well recognized as several studies have suggested that embolic infarcts may affect the posterior cerebral artery in approximately 35% [8] of cases and the cerebellum in up to 54%[9].

The functional anatomy of the cerebellum and thalamus needs to be addressed in order to better understand the spectrum of clinical features that can be seen in stroke involving these structures.

Cerebellar pathology commonly shows important motor signs and less evident cognitive dysfunction. But various studies [10] have strengthened the hypothesis of modulation of cognitive function by the cerebellum and a spectrum of features has been described in the Cerebellar Cognitive Affective Syndrome [10]. This is characterized by impairment of executive function, spatial cognition, language and behavior. The cause is hypothesized to be due to disruption of the circuits linking prefrontal, posterior parietal, superior temporal and limbic cortices of the cerebellum. This reasoning is strengthened by anatomical, physiological and functional neuroimaging studies. Cerebellar infarcts can be classified as territorial and nonterritorial. A better understanding of the variations in the clinical picture is possible when looking at the arterial supply of cerebellum [11]. This is mainly by three long circumferential arteries: the posterior inferior cerebellar arteries, supplying the posterior inferior surface, the anterior inferior cerebellar arteries for the rostral surface and the superior cerebellar arteries which supply the tentorial surface.

Dysarthria and ataxia are prominent in infarcts involving the area supplied by the superior cerebellar artery, whereas lesions involving the inferior cerebellar artery, especially the posterior inferior cerebellar artery, lead to vertigo, headache and vomiting [12]. The other important clinical presentation of posterior inferior cerebellar territory infarcts, of relevance to the case in discussion, is the neuropsychiatric manifestations.

Similarly the thalamic blood supply [11] is derived from four groups of arteries, namely thalamic-subthamic arteries, polar arteries, thalamogenicular arteries and medial and lateral posterior choroidal arteries. Infarcts in the thalamus produce a wide range of clinical syndromes depending on the localization of lesions. It has also been reported that involvement of specific areas of thalamus like the anteromedian territories are predominantly associated with neuropsychiatric manifestations as seen in our case. The most frequent cause of anteriomedial thalamic stroke is cardioembolic [13].

Therefore behavioral and cognitive problems are recognized manifestations of both cerebellar and thalamic infarcts involving the anterior or paramedian nucleus.

Sometimes encephalitis if relatively focal in distribution can look exactly like an infarct on neuroimaging. The typical appearance of encephalitis with the involvement of the medial temporal lobes is sometimes difficult to differentiate and occasionally cerebritis can also mimic infarction [14].

The clinical picture in our patient though still manifesting within the spectrum of features of cerebellar with posterior inferior cerebellar artery territory and thalamic stroke involving the anterior nucleus was difficult to correlate with the diagnosis. This was because the cognitive impairment overshadowed, and was the predominant clinical manifestation, with there being minimal motor impairment. The other differential diagnoses that need to be ruled out by appropriate investigations are meningitis, encephalitis and vasculitis.

## Conclusion

The clinical picture in our patient was neuropsychiatric manifestations without any focal deficits. The symptoms of confusion, memory impairment and personality changes pointed more towards a non-focal neurological cause. After a systematic evaluation, the diagnosis of posterior fossa stroke due to paradoxical embolism was made. This case highlights the importance of thorough evaluation, to rule out systemic causes, while at the same time bearing in mind that it could be an atypical presentation of stroke, especially involving the cerebellum and thalami. The investigations should be directed towards evaluating the etiology in a stepwise manner, to optimise patient care and outcome.

## Abbreviations

Anti-neutrophil cytoplasmic antibody (ANCA), Glasgow coma scale (GCS), Magnetic resonance imaging (MRI), Computed tomography (CT), Patent foramen ovale (PFO)

## Competing interests

The author(s) declare that they have no competing interests.

## Authors' contributions

All authors have contributed equally and have given approval of the version to be published.

## Consent

Written informed consent was obtained from the patient for the publication of the case report and accompanying images. A copy of the written consent is available for review by the Editor-in Chief of this journal.
